# Semaglutide in renal ischemia-reperfusion injury in mice

**DOI:** 10.25122/jml-2022-0291

**Published:** 2023-02

**Authors:** Al-Tameemi Tiba, Heider Qassam, Najah Rayish Hadi

**Affiliations:** 1 Department of Pharmacology and Therapeutics, Faculty of Medicine, University of Kufa, Kufa, Iraq

**Keywords:** ischemia, reperfusion, perfusion reoxygenation, concomitant reoxygenation, PI3K/AKT pathway

## Abstract

Ischemia and reperfusion injury (I/R) is a serious condition leading to organ failure, characterized by poor blood supply followed by rapid resuscitation of blood flow and reoxygenation. Renal failure caused by renal ischemia has high mortality and morbidity. This study aimed to explore the potential role of Semaglutide as a novel and effective therapeutic strategy for acute renal failure. Additionally, we aimed to assess the possible protective effect of Semaglutide on kidney I/R injury in mice through modulation of the inflammatory and oxidative pathways via phosphatidylinositol 3-kinase/adenosine triphosphate (PI3K/AKT) activation. We employed twenty-eight albino mice to induce the I/R injury model by clamping the renal artery for 30 min followed by a period of reperfusion for 2 hours. The control group was exposed to I/R injury, while the Semaglutide-treated group was pretreated with the drug 12 hours before induction of ischemia at a dose of 100 nmol/L/kg via the intraperitoneal route (i.p). In addition, the DMSO-treated group was subjected to similar conditions to the Semaglutide-treated group. At the end of the experiments, kidneys and blood samples were collected for investigation. Semaglutide could act as a protective agent against acute kidney injury by reducing inflammatory molecules such as tumor necrosis factor-alpha (TNF-α) and its cognate receptor, TNF-α R, interleukine-6 (IL-6). Furthermore, Semaglutide reduced F8 isoprostane levels, increased PI3K and AKT levels in renal tissues, and mitigated renal damage. Semaglutide had renoprotective effects via modulation of the inflammatory response and oxidative pathway by targeting the PI3K/AKT signaling pathway.

## INTRODUCTION

Ischemia and reperfusion injury (I/R) of the kidney is a growing public health concern worldwide, particularly in low-income and middle-income countries. It occurs following a period of poor blood supply followed by rapid resuscitation of blood flow and reoxygenation, which can cause organ damage [1]. The causes of renal I/R injury can include sepsis, infarction, renal transplantation, and unilateral nephrectomy [2].

Several signaling pathways contribute to renal damage following I/R, increasing morbidity and mortality [3]. The PI3K-Akt signaling pathway has emerged as an area of interest among the various molecular mechanisms involved in the pathogenesis of renal ischemia and reperfusion injury. PI3K-Akt axis plays a critical role in multiple biological activities such as inflammation, oxidative stress, and chemotaxis; hence, it can be a key pathway in regulating the biological responses against acute kidney injury [4]. Furthermore, PI3K-Akt signaling has been found to be activated following I/R, resulting in increased proliferation and cell viability in renal tubules [5]. Semaglutide is a well-known antihyperglycemic agent used for treating type 2 diabetes, which binds to the glucagon-like peptide (GLP-1) receptor, resulting in increased insulin release and body weight reduction [6]. However, it is uncertain whether Semaglutide has a role in the PI3K-Akt signaling pathway involved in acute kidney injury following I/R. Therefore, this study aimed to investigate the potential renoprotective effects of Semaglutide against renal I/R.

## MATERIAL AND METHODS

A total of twenty Swiss albino male mice, aged 17-18 weeks and weighing 30-40g, were randomly assigned into four groups, with five mice in each group.

The first group was the sham group, where mice were anesthetized with ketamine and xylazine and underwent laparotomy without clamping. The second group was the I/R group, in which mice were anesthetized and subjected to bilateral renal ischemia for 30 min and 2 h reperfusion by clamping and releasing the renal arteries, respectively.

The third group received a dimethyl sulfoxide (DMSO) (vehicle for Semaglutide) injection via the intraperitoneal route (i.p.) 12 hours before ischemia. These mice underwent laparotomy under anesthesia, followed by bilateral renal arteries clamping for 30 min and 2 hours of reperfusion by releasing the clamps. The fourth group was pretreated with Semaglutide at a dose of 100 nmol/L/kg i.p 12 hours prior to the induction of ischemia and reperfusion. At the end of the experiments, the mice were sacrificed, and blood samples were taken directly from the heart to measure the serum levels of tumor necrosis factor-alpha (TNF-α), interleukin-6 (IL-6), TNF-α receptor, urea, creatinine, and tissue levels of PI3K/AKT and F8-isoprostane. The kidneys were fixed in a 10% formalin solution for further investigation to assess the severity of kidney tissue damage.

### Preparation of samples

#### Blood and tissue sampling

At the end of the experiment, blood was collected from the heart, and enzyme-linked immunosorbent assay (ELISA) was used to measure TNF-α, TNF-α R, IL-6, urea, and creatinine in the serum according to the manufacturer’s instructions. Blood samples were centrifuged at 3000 rpm for 10 min without the use of an anticoagulant to prepare the serum. Kidneys were washed with phosphate buffer saline (PBS) and weighted in a ratio of 1 to 10 W/V to prepare homogenates. The homogenization was carried out using a high-intensity ultrasonic liquid processor with PBS containing 1% of protease inhibitor cocktail and 1% of Triton X-100 [7].

#### Tissue sampling for histopathology scoring

The removed kidney was fixed in 10% formalin, processed using standard histological procedures, and embedded in a paraffin block [7]. Kidney tissues were sliced to a thickness of five micrometers and stained with hematoxylin and eosin (H&E). The tissue sections were investigated by an independent histopathologist using a benchtop microscope. The severity of kidney damage involving changes in the tubular cell, such as necrosis, hypertrophy, glomerular atrophy, and inflammatory cell infiltration, was used to identify the extent of the renal damage. Scores were assigned for these variables: (zero) for the sham group considered normal and not subjected to I/R, 25% for mild damage, 50% for moderate damage, 75% for severe damage, and 75%-100% for extremely severe damage [8].

### Statistical analysis

Statistical analysis was conducted using GraphPad Prism 8 software, and the data were presented as mean ± SEM (standard error of the mean). For multiple comparisons, an analysis of variance (ANOVA) followed by Tukey's test was performed. A significance level of P < 0.05 was considered statistically significant.

## RESULTS

### Effects of renal I/R on renal function

**Figure 1:**
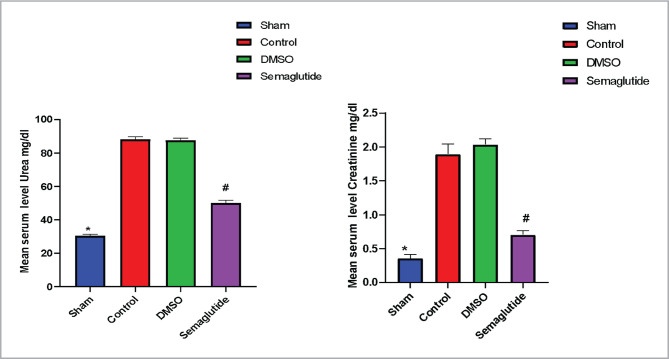
The mean serum level of urea and creatinine (mg/dl) in the four experimental groups, data are expressed as mean ± SEM; *P<0.05 sham vs. control; #P<0.05 Semaglutide vs. I/R mice.

Figure 1 shows that serum urea and creatinine levels were significantly elevated in the control and DMSO groups compared to the sham group. However, in the Semaglutide pretreatment group, these levels were significantly reduced, indicating that Semaglutide may have protective properties against renal I/R injury.

### Semaglutide inhibits tissue levels of TNF-α, TNF-α R, and IL-6

**Figure 2:**
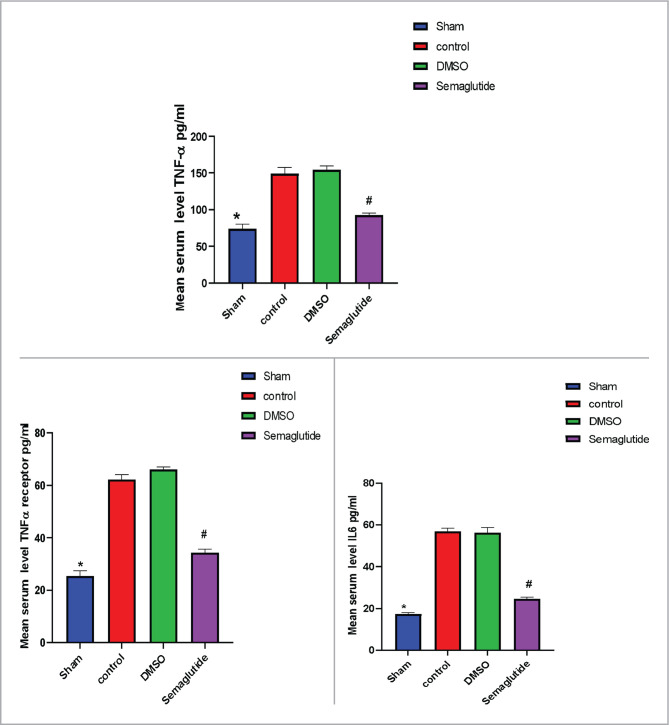
The mean serum level of TNF-α, TNF-αR, and IL-6 (pg/ml) among four experimental groups. Data are expressed as mean ± SEM; *P<0.05 sham vs. control; #P<0.05 Semaglutide vs. I/R mice.

The impact of renal I/R on inflammatory mediators was assessed in different groups. The serum levels of TNF-α, TNF-α R, and IL-6 were significantly increased (p<0.05) in the control group as compared to the sham group (Figure 2). In contrast, these levels were significantly decreased in the Semaglutide-treated group (Figure 2), indicating that Semaglutide may have anti-inflammatory effects in the context of renal I/R injury.

### Semaglutide reduces F8 isoprostane (F8IsoP)

**Figure 3:**
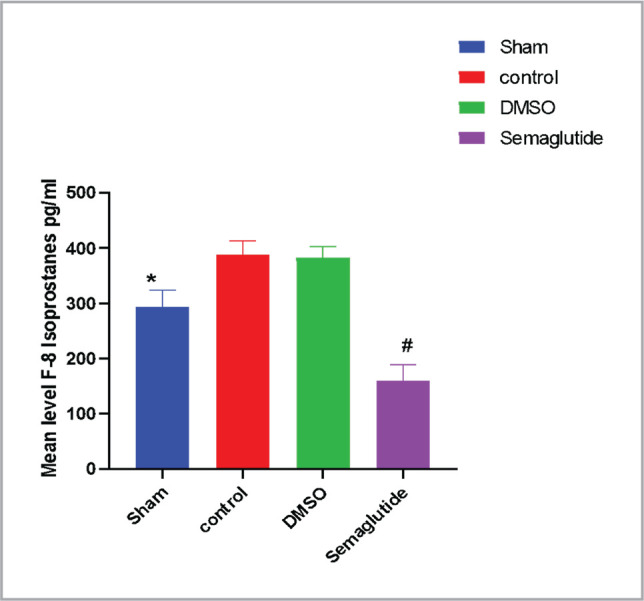
The mean level of tissue F8 ISOP (pg/ml) in the four experimental groups, data are expressed as mean ± SEM; * P<0.05 sham vs. control; # P <0.05 Semaglutide vs. I/R mice.

F8 isoprostane (F8IsoP) is a widely recognized biomarker of oxidative stress and is considered a critical indicator of organ injury. As shown in Figure 3, the levels of F8IsoP in the I/R group were significantly higher than those in the sham group, indicating increased oxidative stress in the kidneys following I/R injury. However, treatment with Semaglutide led to a marked reduction in F8IsoP levels, suggesting that Semaglutide may have protective effects against oxidative stress in the context of renal I/R injury.

### Phosphatidylinositol-3-kinase in a polymerase chain reaction (PCR)

**Figure 4:**
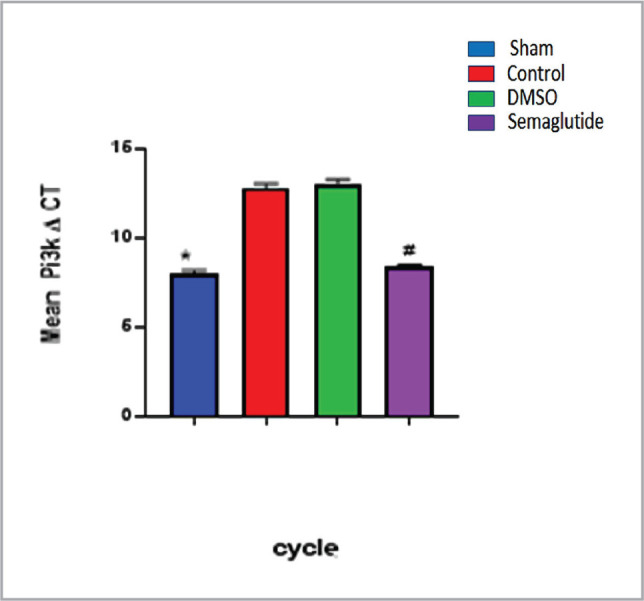
The mean expression level of renal tissue PI3K in the four experimental groups. Data are expressed as mean ± SEM; * P<0.05 sham vs. control; # P<0.05 Semaglutide vs. I/R mice.

To investigate the expression of PI3K in renal tissue, PCR analysis was performed, and the results are shown in Figure 4. The levels of PI3K were significantly higher in the control group than in the sham group, as indicated by ∆CT values. However, treatment with Semaglutide resulted in a marked reduction in PI3K expression levels, with values returning to those observed in the sham group (p<0.05) (Figure 4).

### Immunohistochemistry study

**Figure 5:**
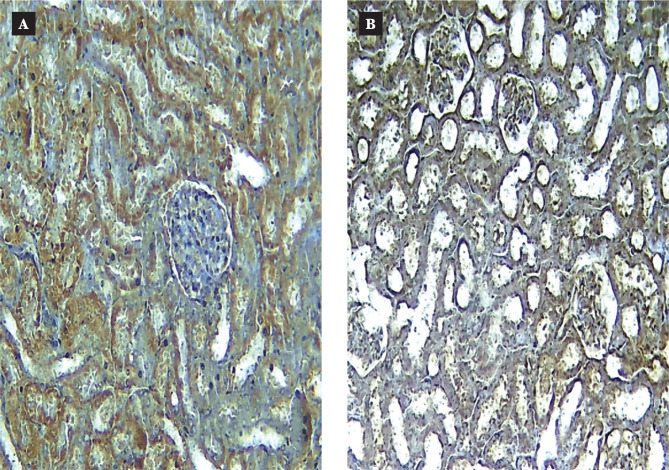

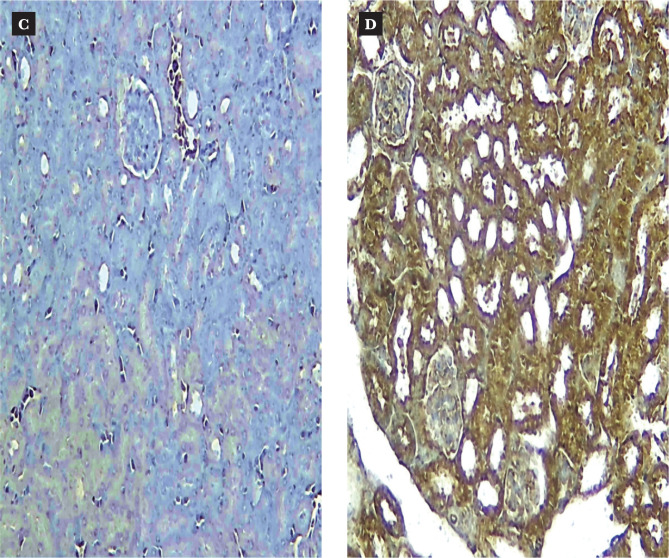
Data in are expressed as mean ± SEM; * P<0.05 sham vs. control; #P<0.05 Semaglutide vs. I/R mice. A: Representative Akt immunohistochemistry (IHC) staining in renal tissue of mice in the sham group. The result shows the normal expression level of Akt in renal tissue; B: Representative Akt IHC staining in renal tissue of mice in the control group. The result shows the low expression level of this protein in renal tissue; C: Representative Akt IHC staining in renal tissue of mice in the DMSO group. The result shows the low expression level of this protein in renal tissue; D: Representative Akt IHC staining in renal tissue of the Semaglutide group. The result shows the high expression level of this protein in renal tissue.

The immunoreactivity of Akt antibody was examined to investigate Akt protein expression in renal tissue following I/R injury. As shown in Figure 5, the Akt protein levels were significantly lower in the I/R injury group than in the sham group (p < 0.05), indicating reduced Akt protein expression in response to I/R injury. However, treatment with Semaglutide resulted in a significant increase in Akt protein levels compared to the I/R injury group (p < 0.05), as depicted in Figure 5 A–D.

### Effects of semaglutide on renal histology

**Figure 6:**
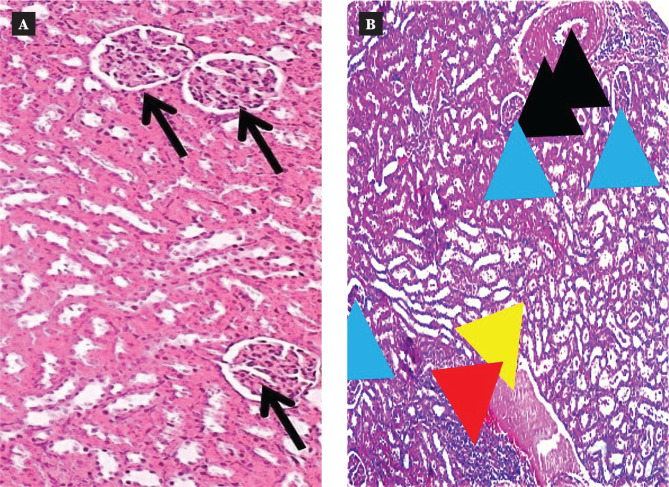

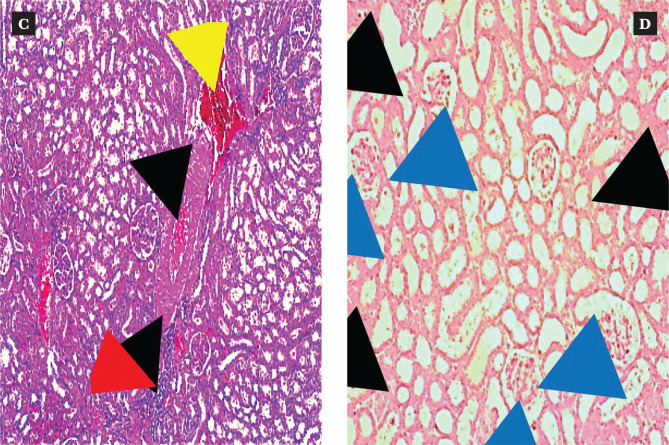
A: The histopathological section of renal tissue in the sham group shows normal glomerular texture (black arrows). The tissue was stained using H&E stain and examined using a light microscope and digital camera at a 10X magnification scale. B: The histopathological section of renal tissue in the control group (I/R) shows clear congestion in the blood vessels (black arrow), thickening of the blood vessel wall, and reduced glomerular size (glomerular atrophy, blue arrows). The section shows the infiltration of pinkish structures and homogenous material (amyloid degeneration, yellow arrow) as well as the infiltration of inflammatory cells, primarily neutrophils, in the renal tissue (red arrow). The tissue was stained using H&E stain and examined using a digital camera and light microscope at a 10X magnification scale. C: The histopathological section of renal tissue in the DMSO group shows clear congestion in the blood vessels (black arrow) and thickening of the blood vessel wall. The section shows inflammatory cell infiltration, mainly neutrophils in the renal tissue (red arrow), and there is apparent hemorrhage in the renal tissue near the blood vessels (yellow arrow). The tissue was stained using H&E stain and examined using a digital camera and light microscope at a 10X magnification scale. D: The histopathological examination of renal tissue in the Semaglutide group shows mild hypertrophy of renal tubules (black arrows) and mild atrophy of the renal glomeruli with an increase in the Bowman's space (blue arrows). The tissue was stained using H&E stain and examined using a light microscope and digital camera at a 10X magnification scale.

**Figure 7:**
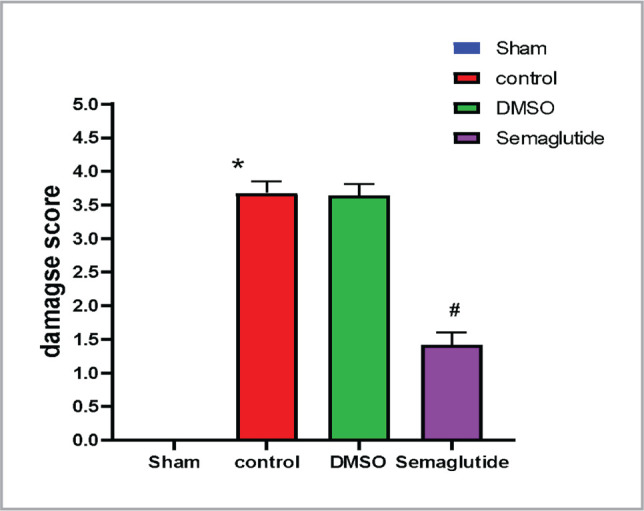
Renal damage scores in the four groups were examined and scored according to a protocol previously described [8]. All data are expressed as Mean ± SEM (n=5), P≤0.05 versus sham, # P≤0.05 Semaglutide versus I/R mice.

The histological analysis of renal tissue following I/R injury revealed severe damage characterized by congestion, thickening of blood vessels, inflammation, and hemorrhage, as compared to the normal structures observed in the sham group (Figures 6 A–D and 7, Table 1). However, treatment with Semaglutide resulted in a significant reduction in the severity of renal damage, as evidenced by a lower damage score (p < 0.05), as shown in Figures 6 A–D and 7, Table 1.

**Table 1:** The differences in histopathological scoring among the four experimental groups.

Histological grading	Sham		Control		DMSO		Semaglutide	
	N	%	N	%	N	%	N	%
**No abnormality (0)**	5	100	0	0	0	0	0	0
**Mild (1)**	0	0	0	0	0	0	1	20
**Moderate (2)**	0	0	0	0	0	0	4	80
**Severe (3)**	0	0	1	20	2	40	0	0
**Highly severe (4)**	0	0	4	80	3	60	0	0
**Total**	5	100	5	100	5	100	5	100

## DISCUSSION

Acute kidney injury is a significant clinical problem that affects populations worldwide and is associated with high morbidity and mortality rates. Despite advances in understanding the pathogenesis and biomarkers involved in acute kidney injury, there is currently limited knowledge about the fundamental mechanisms behind the deterioration of renal function following injury [9]. Renal I/R is one of the critical players that contribute to the development of acute kidney injury via its role in various biological responses, including inflammation, cell apoptosis, and free radical accumulation [10].

The present study found that levels of urea and creatinine in the serum were elevated in mice subjected to renal I/R compared to the sham group, consistent with previous research indicating that 30 min of ischemia followed by 2 h of blood resuscitation increased levels of creatinine and alanine aminotransferase [11]. These are critical indicators of renal function that are elevated in response to injury. In contrast, treatment with Semaglutide resulted in a marked decrease in levels of serum urea and creatinine in comparison with the renal I/R injury group suggesting that the drug has a protective influence against kidney injury. These findings are consistent with other research indicating that GLP-1 receptor agonists may have renoprotective effects through diuretic and natriuretic properties, as well as a reduction in renal angiotensin II [12].

The results of this study indicate that serum levels of IL-6, TNF-α, and TNF-α receptors were significantly increased in mice exposed to I/R compared to the sham group. These findings are consistent with earlier studies that reported increased levels of these biomarkers in response to I/R injury [13,14]. It is well known that I/R injury leads to many events, including inflammatory responses characterized by increased inflammatory cytokines and macrophage recruitment leading to renal damage [15]. Treatment with Semaglutide reversed the earlier results and reduced these markers in comparison with the I/R group. In accordance with the present results, previous studies have demonstrated that activating GLP-1 receptor by Liraglutide markedly reduced expression of IL-6, TNF-α, TLR2, and TLR4, and mitigated the renal damage and ameliorated the kidney function suggesting that GLP-1 receptor agonists may be potential candidates against renal injury [16].

In this study, we observed a significant increase in levels of F8-isoprostane in the mice subjected to I/R injury compared to the sham group. This finding is consistent with previous studies that have reported F8-isoprostane as a critical biomarker of oxidative damage and an important contributor to tissue injury following I/R injury [17]. In contrast, the treatment with Semaglutide significantly decreased the levels of F8-isoprostane in the renal tissues, indicating a potential impact on oxidative stress. Previous research has shown that GLP-1 receptor activation can reduce platelet activity, a critical factor in lipid peroxidation and inflammation. Therefore, GLP-1 receptor agonists like Semaglutide may exert antioxidant effects [18].

The most notable finding from this study was the significant reduction in PI3K expression levels in the renal tissues of the I/R injury group compared to the sham group, as indicated by ∆CT values (Figure 4). These findings seem consistent with other research, which revealed that I/R resulted in a modulation in PI3K/Akt signaling by decreasing levels of PI3K expression in the kidney [19]. By contrast, treatment with Semaglutide markedly increased levels of PI3K expression and preserved these levels close to the sham group. These results are consistent with recent studies indicating that activating the GLP-1 receptor can reverse the decreased levels of both PI3K and Akt in rat models of I/R injury, suggesting that GLP-1 may play a crucial role in promoting cell survival via modulating the PI3K/Akt axis [20]. This signaling pathway is essential in maintaining renal function, as documented in previous studies [15, 21]. It has inhibitory influences on a variety of biomarkers, such as proinflammatory cytokines and apoptotic molecules that contribute to the pathogenesis of renal injury [22].

The current study revealed that mice subjected to I/R had significantly higher levels of renal tissue damage when compared to the sham group. These findings are consistent with previous studies that reported similar features of renal tissue damage following I/R injury, including tubular cell swelling, pyknotic nuclei, cellular vacuolization, congestion, and cellular necrosis [23,24]. In contrast, treatment with Semaglutide notably reduced the degree of damage in the renal tissues suggesting its renoprotective impact. The results of this research support earlier observations, which showed that Semaglutide mitigated renal injury and reduced the glomerulosclerosis index in a mouse model of hypertension-induced diabetic kidney disease [25].

## CONCLUSION

The findings of this study suggest that Semaglutide may have renoprotective effects by inhibiting inflammatory and oxidative stress mediators and activating the PI3K/AKT pathway. Further studies are needed to fully elucidate the mechanisms behind these effects and to evaluate the clinical application of Semaglutide in the treatment of kidney injury.
